# Knowledge, attitudes and practices of antimicrobial resistance awareness among healthcare workers in India: a systematic review

**DOI:** 10.3389/fpubh.2024.1433430

**Published:** 2024-07-22

**Authors:** Shweta Rana, Karuna Nidhi Kaur, Priyanka Narad, Kamini Walia, Shazina Saeed, Amrish Chandra, Mohd Shannawaz, Harpreet Singh

**Affiliations:** ^1^Division of Development Research, Indian Council of Medical Research (ICMR), New Delhi, India; ^2^Amity Institute of Public Health and Hospital Administration (AIPH&HA), Amity University, Noida, India; ^3^Division of Descriptive Research, Indian Council of Medical Research (ICMR), New Delhi, India; ^4^School of Pharmacy, Sharda University, Greater Noida, India

**Keywords:** antimicrobial resistance, knowledge, attitudes, practices, healthcare workers, India

## Abstract

**Objectives:**

The study was conducted to identify and compile gaps in the Knowledge, Attitudes, and Practices (KAP) regarding Antimicrobial Resistance (AMR) among healthcare workers in India.

**Methods:**

A systematic review of published literature from PubMed, Google Scholar, and Scopus databases was conducted in compliance with the PRISMA guidelines. The inclusion criteria focused on studies evaluating KAP toward AMR among various healthcare workers in India without restricting context to specific diseases. We included articles published from inception to December 2023.

**Results:**

Following the inclusion criterion, 19 studies were selected for the review. The study has a cumulative sample size of 4,544 healthcare providers across India. We found that doctors and medical students have significant knowledge about AMR, followed by nurses and pharmacists. However, the attitudes toward AMR were higher among informal providers, followed by doctors and medical students. The study also observed a gap between theoretical knowledge and practical application of AMR principles among healthcare providers in India.

**Conclusion:**

The study highlights the need for targeted training and policy interventions to bridge the gap between KAP regarding AMR. Healthcare providers can significantly contribute to mitigating AMR threat by improving KAP related to AMR. This systematic review provides a foundation for developing and implementing effective evidence-based strategies to enhance AMR containment in India.

## Introduction

Antimicrobial Resistance (AMR) is a multifaceted global challenge to the public health. Rising AMR has significant health and economic consequences (1). The impact of AMR is more prominent in developing countries due to higher infection rates, inappropriate use of antibiotics, poor drug quality and access, lack of regulation and surveillance and socioeconomic factors (2, 3). The consequences of increasing AMR included 1.27 million deaths worldwide in 2019 (4). According to the report, By 2050, it is estimated that AMR will be responsible for approximately 10 million deaths annually worldwide. Of these, 9 million deaths will be from developing countries, including 4.7 million in Asia. India alone will contribute to 2 million deaths annually, with an economic burden of as much as $100 trillion (5, 6). The risk assessments conducted by the World Health Organization (WHO) found that the Southeast Asia region, including India, is the most at-risk part of the world for AMR (7–9).

Many factors are contributing to rising AMR in India. These include unregulated antibiotic access, including over-the-counter sales without prescription, incompatible infection prevention control (IPC) practices, antibiotic use and misuse, and lack of stringent guidelines (10, 11). A common cause that can explain most risk factors is awareness of AMR among different stakeholders. Knowledge, Attitudes and Practices (KAP) is one of the most accepted techniques for evaluating stakeholder awareness. Though there are several KAP studies worldwide, only a handful are from India. The studies independently highlight that a significant number of individuals lack comprehension regarding the consequences of prematurely discontinuing a course of antibiotics or employing them without a legitimate need. This behavior can result in the development of antibiotic-resistant microorganisms (12). Healthcare workers can significantly reduce AMR and ensure that medications remain effective through knowledge, appropriate attitudes and practices toward antibiotic prescriptions (13, 14). It further requires a combined effort from different strata of health professionals, including doctors, nurses, pharmacists, and informal providers, to rationally prescribe antibiotics and promote patient awareness.

Large populations and limited health resources are crucial factors in developing countries such as India that result in improper antibiotic use (2, 15). Reducing antibiotic consumption is critical to preventing the spread of AMR and can be achieved through changes in prescribing practices and improved knowledge and attitudes toward antibiotic resistance. Evidence-focused questionnaires such as KAP surveys can analyze factors influencing doctors’ prescribing behaviors (16, 17).

To the best of our knowledge and understanding, this is the first systematic review and descriptive analysis that integrates and explores the KAP of healthcare professionals, encompassing medical students, physicians, dentists, pharmacists, nurses and informal providers in India toward AMR. The review will be a valuable resource for healthcare professionals aiming to enhance antibiotic prescription practices and assist policymakers in designing evidence-based strategies to combat antibiotic resistance in India. The challenge of AMR in India requires a comprehensive, coordinated, and multifaceted approach.

This review was conducted to comprehensively investigate and compile the existing KAP surrounding AMR among healthcare workers in India. By systematically reviewing available literature, we aim is to identify gaps, patterns, and disparities in the understanding, beliefs, and behaviors related to AMR among various cadres of healthcare professionals, including medical students, physicians, dentists, pharmacists, nurses, and informal providers. This review offers a nuanced understanding of the challenges and opportunities in addressing AMR within the Indian healthcare context. Furthermore, by synthesizing findings, the review aims to suggest targeted interventions, policies, and educational initiatives to mitigate the impact of AMR and promote prudent antimicrobial use practices across diverse healthcare settings in India.

## Materials and methods

### Protocol registration methods

The systematic review protocol was designed in line with the Preferred Reporting Items for Systematic Reviews and Meta-Analyses (PRISMA) 2020 guidelines and prospectively registered with PROSPERO (ID: CRD42023454975) (18, 19).

### Search methods

A literature search was performed across “PubMed,” “Google Scholar,” and “Scopus.” The search strategy included both Medical Subject Headings (MeSH) and keywords such as “Antimicrobial Resistance” OR “AMR” OR “Antibiotic resistance” AND “Knowledge, Attitudes and Practices” OR “KAP” AND “Healthcare Worker” OR “Health Professionals” OR “Health Workers” OR “Clinician” OR “Junior Doctor” OR “MBBS Student” OR “Physician” AND “India.” The keywords terms were combined using Boolean operators to encompass a broad spectrum of relevant studies. Only peer-reviewed and published journal articles were reviewed.

### Eligibility criteria for study selection

The systematic review followed strict inclusion and exclusion criteria to focus on the KAP of AMR among healthcare workers in India. We included original research articles that examined KAP pertaining to AMR without limiting the context to specific diseases, which allowed for a broader understanding of AMR within the healthcare system.

In contrast, studies primarily focused on hospital infection, antibiotic prescribing behavior, and antimicrobial stewardship, or those specific to diseases such as tuberculosis and polio were excluded. We also confined our review to English publications, available until December 2023, which ensured that the selected studies were relevant and accessible to the international academic community. Consequently, any non-English studies, as well as any literature published beyond this date, were excluded. Furthermore, the review excluded non-original research such as review articles, case reports, case series, commentary, and correspondence to ensure a focus on novel, empirical studies that directly address our research questions regarding AMR KAP among Indian healthcare workers.

### Study selection

Two independent reviewers conducted both the initial title and abstract reviews, as well as the subsequent full-text reviews, separately. This approach ensured consistent and impartial application of the inclusion criteria. Any discrepancies between the researchers decision were resolved through thorough discussions, and if consensus could not be reached, a third researcher (Principal Investigator) was consulted to make the final determination. This multi-step review process helped mitigate personal biases and enhance the objectivity of our study selection.

Initially, we reviewed the titles and abstracts of potential studies to determine eligibility. In the second step, we obtained and examined the full texts of these studies for a more in-depth evaluation. Specifically, we included only articles focused on the KAP of AMR among healthcare workers in India. Our initial database search yielded 8,797 published articles matching the specified keywords. After removing 450 duplicate articles, we excluded 6,157 studies that did not focus on AMR among Indian healthcare workers.

**Figure 1:**
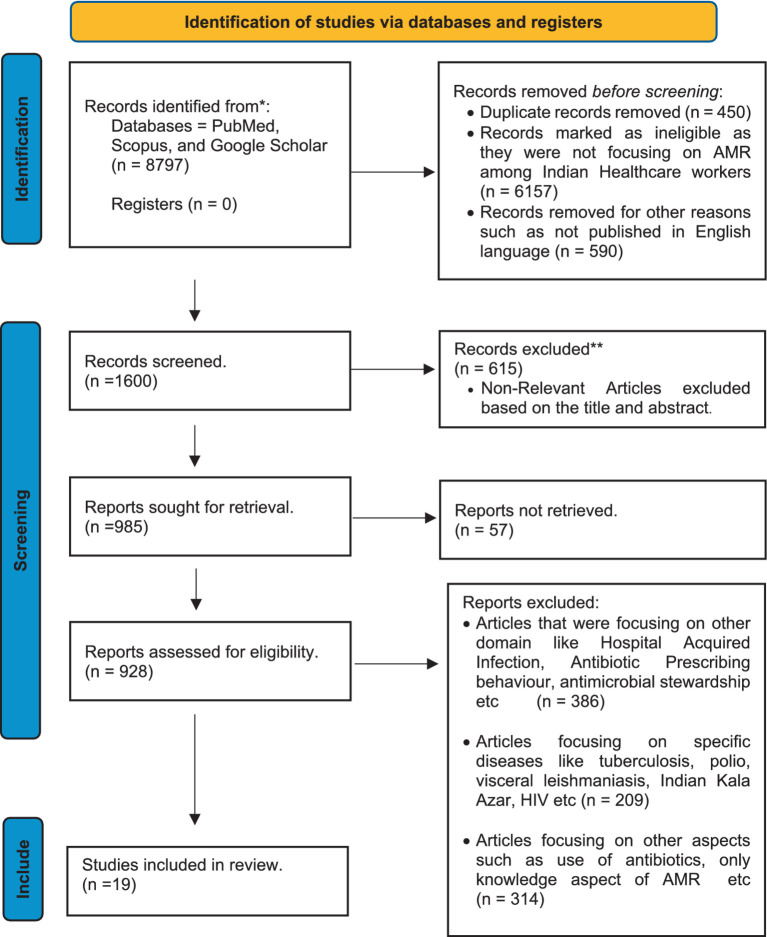
Prisma diagram.

Furthermore, 590 articles were removed due to non-English publications or ineligible articles. Subsequently, 1,600 articles were screened by reviewing either the abstracts or full text, eliminating another 615 articles. After the screening, 985 articles were sought for retrieval, and 57 were unable to be retrieved. Finally 928 articles were assessed for eligibility. Articles excluded that were focused on other domains such as Hospital Acquired Infection, Antibiotic Prescribing behavior, antimicrobial stewardship, etc. (*n* = 386) or specific diseases like tuberculosis, polio, visceral leishmaniasis, Indian Kala Azar, HIV etc. (*n* = 209), focusing on other aspects such as the use of antibiotics, only knowledge aspect of AMR etc. (*n* = 314). Following the final screening and evaluation, 19 articles that met the inclusion criteria were finally included, as illustrated in Figure 1.

### Data extraction

The data were manually extracted and collated into Microsoft Excel spreadsheets, and the following domains of data were extracted from each study: participant’s information (age, gender), sample size, country/State/City, knowledge, attitudes and practices percentage, outcome measures and findings. Studies were categorized and compared based on the KAP score. Given the nature of the review question, a meta-analysis was not undertaken, and instead, a descriptive synthesis and thematic analysis were done.

### Risk of bias assessment

The bias assessment in this systematic review was conducted using the R-based Robvis software package, a tool introduced by the National Institute for Health Research (NIHR) to visualize risk-of-bias assessments (20). We followed the Cochrane Risk of Bias Tool to evaluate the included studies, which encompasses several domains to assess potential sources of bias. Selection bias was assessed by evaluating the methods used to generate and conceal the allocation sequence, ensuring no manipulation in the allocation of participants and no systematic differences in baseline characteristics. Performance bias was examined by checking whether participants and personnel were blinded to the intervention allocation, thus preventing behavior or performance differences that could influence outcomes. Detection bias was assessed by determining if outcome assessors were blinded to the intervention allocation, ensuring impartial measurement of outcomes. Attrition bias was evaluated by examining the completeness of outcome data, looking for systematic differences in withdrawals or exclusions between study arms. Reporting bias was assessed by checking for selective outcome reporting, ensuring that all pre-specified outcomes were reported accurately. Each study was evaluated across these domains, with the risk of bias categorized as low, unclear, or high.

**Figure 2:**
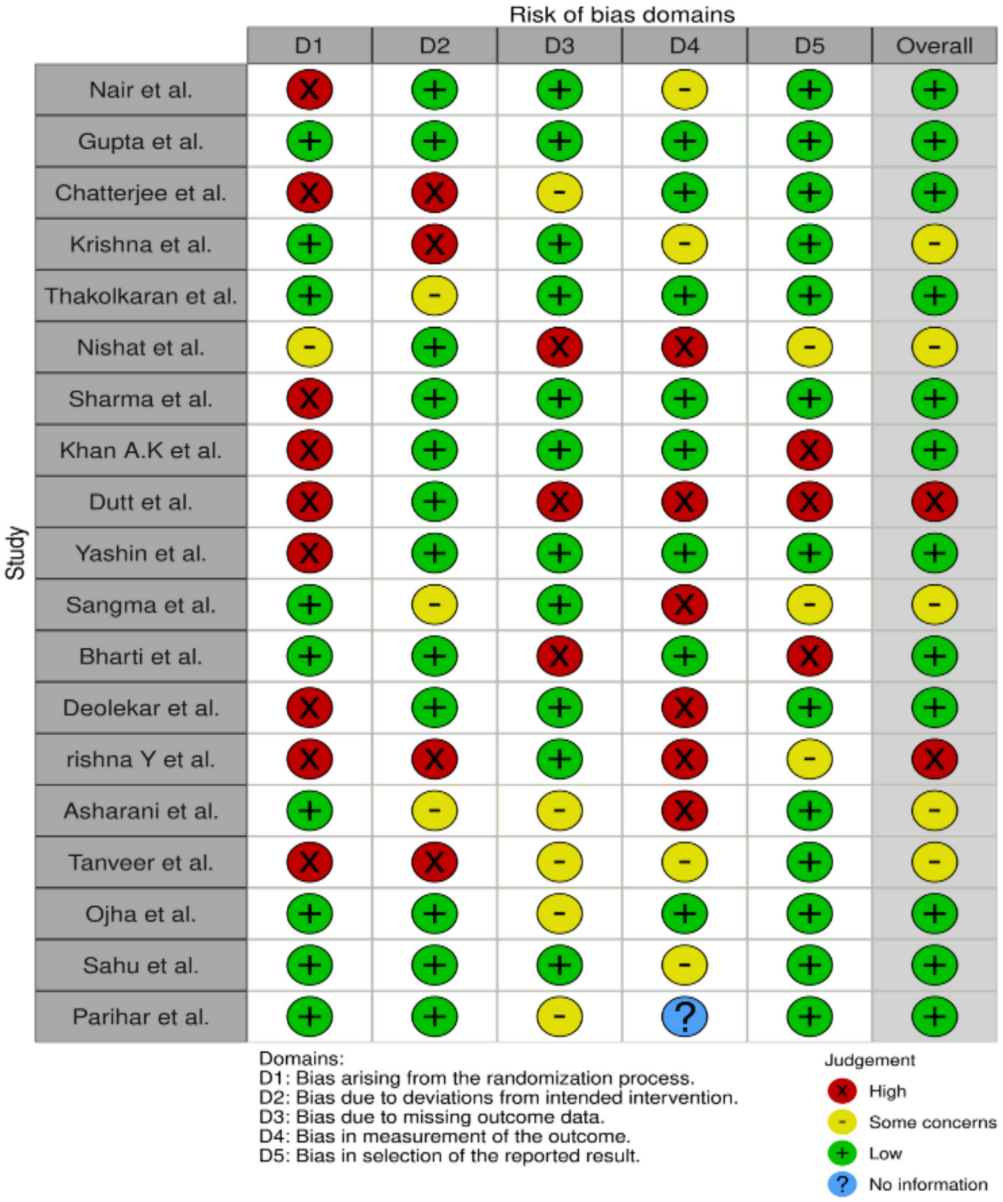
Weighted output for risk bias assessment.

**Figure 3:**
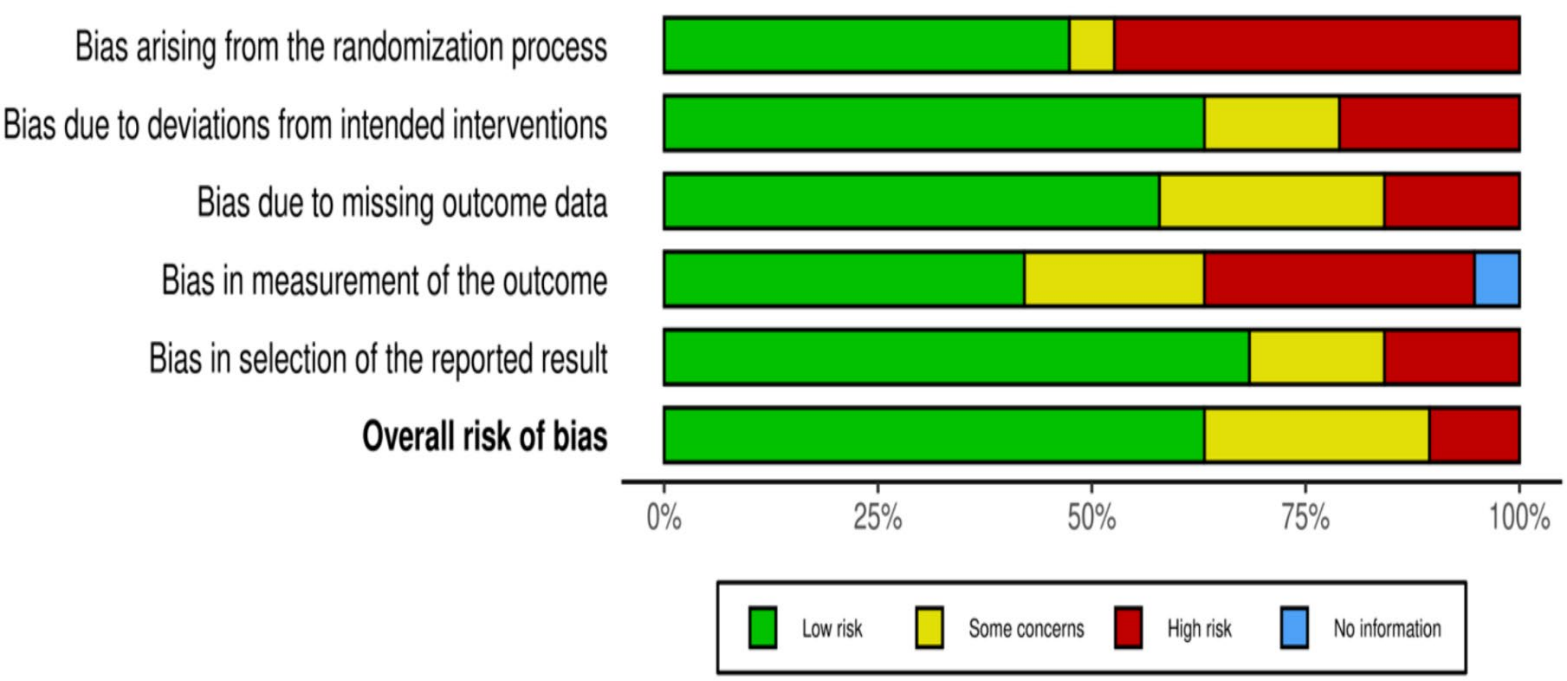
Robvis output for risk bias assessment.

The outcomes of the risk of bias evaluation are depicted in Figures 2, 3, which indicate an absence of potential bias. The majority of included studies (17 out of 19) exhibited high methodological quality with a low risk of bias. These studies supports the validity of the observed gaps in KAP regarding AMR among healthcare workers in India and adhered closely to the rigorous research standards, reinforcing the reliability of our systematic review’s findings. Only two studies exhibited a high risk of overall bias, as depicted in Figure 2.

## Results

### Main characteristics of the included studies

A total of 19 studies were included, and the key features are detailed in Table 1. The studies span across various states in India, with the majority of studies conducted in West Bengal, Karnataka, and Kerala. Geographically, the regions with the highest number of studies are Kerala (*n* = 4) and Karnataka (*n* = 4), representing a significant portion of the data. All studies were published over a period of 11 years, from 2013 to 2023, encompassing diverse healthcare environments and professional demographics. There was substantial variation in the duration of these studies demonstrating the myriad approaches and scopes within the research methodologies.

**Table 1:** Characteristic table of included studies.

S. No.	Name of author	Year	Study design	Age (in years)	Sample size	Participants details	State	Knowledge (%)	Attitudes (%)	Practices (%)
1	Afzal Khan et al. (21)	2013	Cross-sectional study	19–28 years	97	97 Medical Students	Kerala	82.55%	59.08%	66.59%
2	Krishna et al. (22)	2015	Cross-sectional study	19–30 years	150	150 Medical Students	Andhra Pradesh	96.00%	64.60%	68.70%
3	Sharma et al. (23)	2016	Cross-sectional study	19–28 years	110	110 Medical Students	Kerala	79.72%	55.95%	64.00%
4	Thakolkaran et al. (24)	2017	Cross-sectional study	Mean age-34.46 (8.5)	230	230 Doctors	Karnataka	62.97%	89.00%	74.25%
5	Krishna et al. (25)	2018	Cross-sectional study	30–62 years	118	60 Doctors 58 Pharmacist	Karnataka	71.46%	87.68%	57.96%
6	Dutt et al. (26)	2018	Cross-sectional study	20–40 years	222	222 Medical Students	Kerala	77.50%	79.70%	66.20%
7	Yashin et al. (27)	2018	Cross-sectional study	20–60 years	270	188 Medical Students 82 Doctors	Assam	86.62%	72.33%	65.11%
8	Sangma et al. (28)	2018	Cross-sectional study	21–30 years	167	139 Medical Students 28 Doctors	Imphal	52.69%	52.78%	50.34%
9	Nair et al. (29)	2019	Cross-sectional study	18–77 years	384	96 Doctors 96 Nurses 96 Informal Providers 96 Pharmacists	West Bengal	60.49%	80.21%	72.60%
10	Gupta et al. (30)	2019	Cross-sectional study	18–40 years	474	474 Medical Students	Rajasthan (*n* = 177) Delhi (*n* = 59) Maharashtra (*n* = 42) Uttar Pradesh (*n* = 36) Odisha (*n* = 29) Uttarakhand (*n* = 29) Karnataka (*n* = 20) Madhya Pradesh (*n* = 14) Puducherry (*n* = 13) Gujarat (*n* = 12) Punjab (*n* = 8) Chhattisgarh (*n* = 8) Ladakh (*n* = 5) Bihar (*n* = 5) West Bengal (*n* = 5)	86.86%	61.10%	37.19%
							Andhra Pradesh (*n* = 3) Tamil Nadu (*n* = 3) Haryana (*n* = 2) Kerala (*n* = 2) Meghalaya (*n* = 1) Himachal Pradesh (*n* = 1)			
11	Deolekar et al. (31)	2019	Cross-sectional study	19–28 years	200	200 Medical Students	Maharashtra	89.70%	61.38%	61.71%
12	Parihar et al. (32)	2019	Cross-sectional study	19–24 years	350	350 Medical Students	Jammu & Kashmir	85.50%	76.07%	54.27%
13	Bharti et al. (33)	2020	Cross-sectional study	18–28 years	359	359 Medical Students	Himachal Pradesh	70.95%	74.88%	56.80%
14	Asharani et al. (34)	2020	Cross-sectional study	20–35 years	367	367 Medical Students	Karnataka	77.09%	58.99%	83.45%
15	Sahu and Sahu (35)	2021	Cross-sectional study	20–60 years	100	100 Nurses	Chhattisgarh	61.90%	66.00%	44.11%
16	Nishat et al. (36)	2022	Cross-sectional study	18–55 years	120	120 Medical Students	Telangana	60.00%	61.67%	66.67%
17	Chatterjee et al. (37)	2022	Cross-sectional study	Mean-31.4 ± 8.71	506	506 Doctors	West Bengal (*n* = 114) Chandigarh (*n* = 92) Madhya Pradesh (*n* = 200) Gujarat (*n* = 100)	79.83%	74.57%	65.50%
18	Tanveer et al. (38)	2022	Cross-sectional study	<30 to >40 years	40	40 Pharmacists	Telangana	56.60%	56.43%	29.84%
19	Ojha et al. (39)	2023	Cross-sectional study	20–30 years	280	280 Doctors	Madhya Pradesh	71.80%	53.78%	63.97%

### Demographic of study population

**Figure 4:**
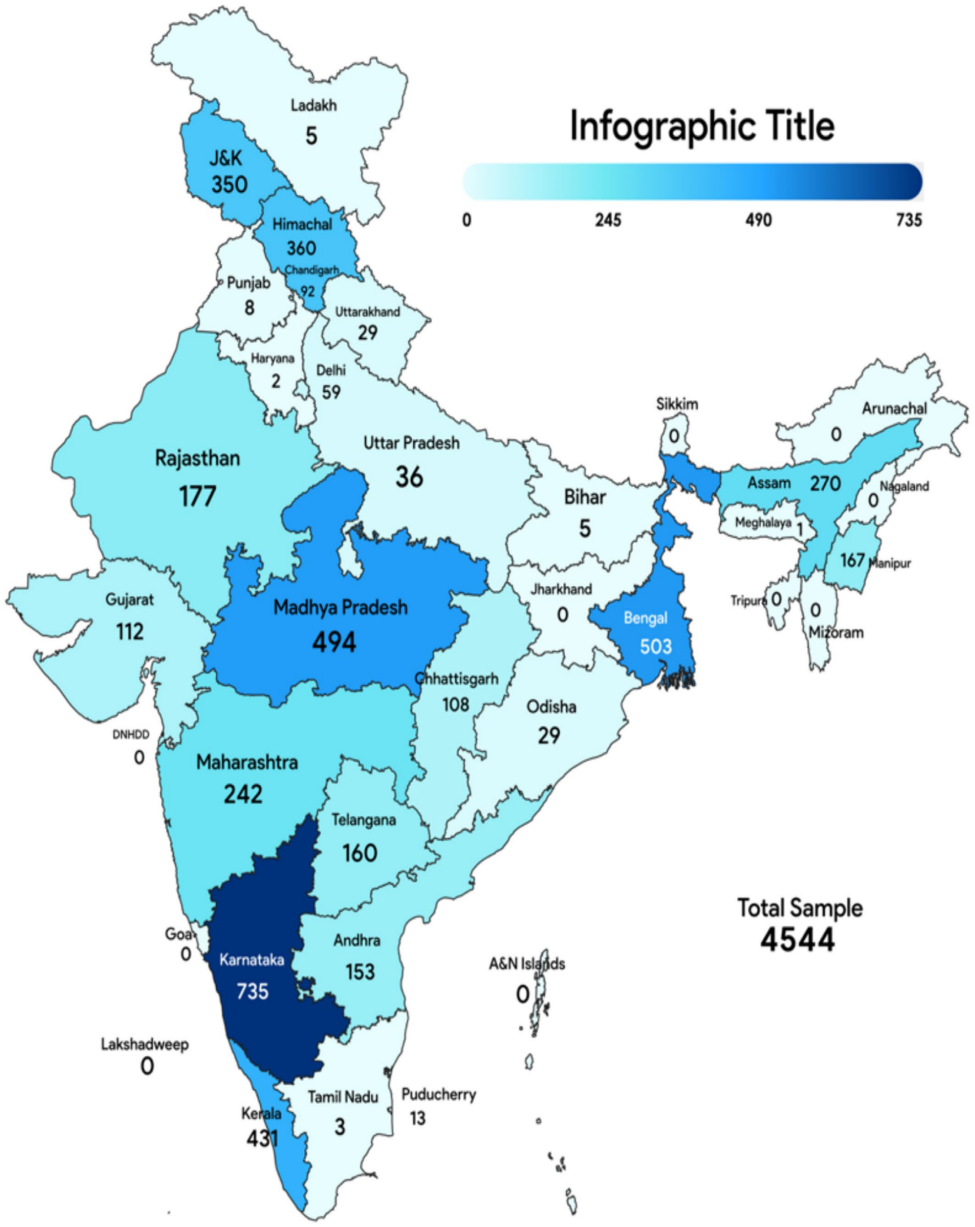
Geographical distribution of included studies.

The review encompasses an analysis of the KAP of 4,544 healthcare professionals in 19 studies across various regions of India, as shown in Figure 4. Of the 19 studies analyzed, gender distribution data were accessible for 14, providing details on a subset of the overall sample. Despite these limitations, the findings revealed an equitable gender representation, with 1,669 male and 1,612 female participants. The participant’s ages range from 18 to 77 years, incorporating both early career professionals and those with extensive experience in the field. This scope provides a comprehensive perspective on the KAP of AMR within healthcare settings.

**Figure 5:**
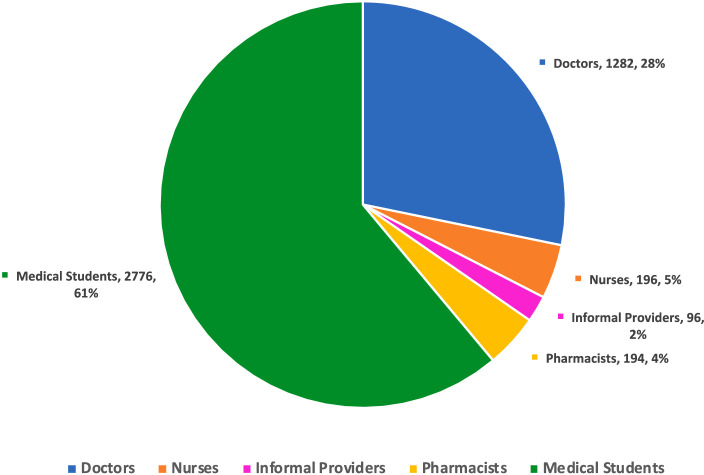
Distribution of healthcare workers in included studies.

Figure 5 depicts the distribution of the participant demographics in the included studies. It shows that most participants were medical students, 61% (*n* = 2,776) of the total sample. Following them, physicians comprise the second-largest group at 28% (*n* = 1,282), indicating a significant inclusion of current prescribers in the study cohort. The data further show that nurses (5%, *n* = 196) and pharmacists (4%, *n* = 194) are less represented, while informal providers are minimally included at 2% (*n* = 96).

**Figure 6:**
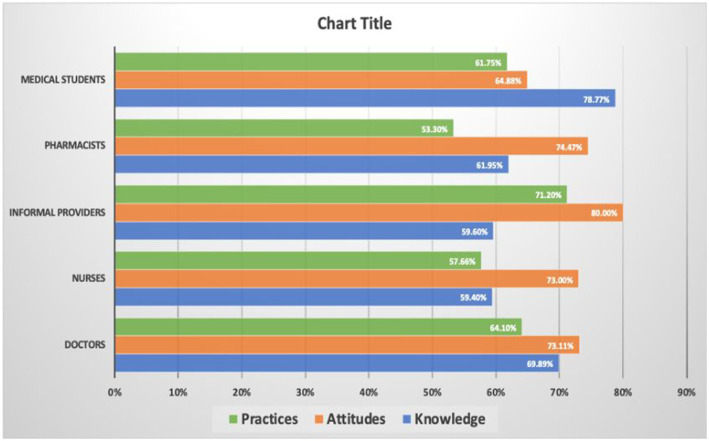
Analysis of knowledge, attitudes, and practices on antimicrobial resistance among healthcare workers.

Figure 6 represents an assessment of various regions on three critical measures concerning AMR: knowledge, attitudes, and practices. It highlights that medical students, followed by doctors, exhibit the highest understanding of AMR. Nurses, pharmacists, and informal healthcare providers show decreased knowledge, respectively. However, the assessment of attitudes shows a reverse trend. Informal healthcare providers show the most positive attitudes, around 80%, toward AMR. Pharmacists and nurses trail closely behind, with doctors and medical students showing lesser degrees of positive attitudes despite their higher knowledge base. For practices, the data suggests an interplay between knowledge, attitudes, and the translation into actual behaviors. Informal healthcare providers exhibit (75%) good practices followed by medical students and doctors. However, good practices to combat resistance sometimes correlate with higher knowledge. While not as knowledgeable, informal healthcare providers often show better practices influenced by their positive attitudes, implying that effective measures against AMR require education and fostering attitudes that encourage proper practices.

This study employed a targeted approach to assess regional disparities in knowledge, awareness and practices concerning AMR among healthcare professionals across India. To ensure the specificity and accuracy of our comparative analysis, we excluded studies that lacked detailed KAP data for each state.

**Figure 7:**
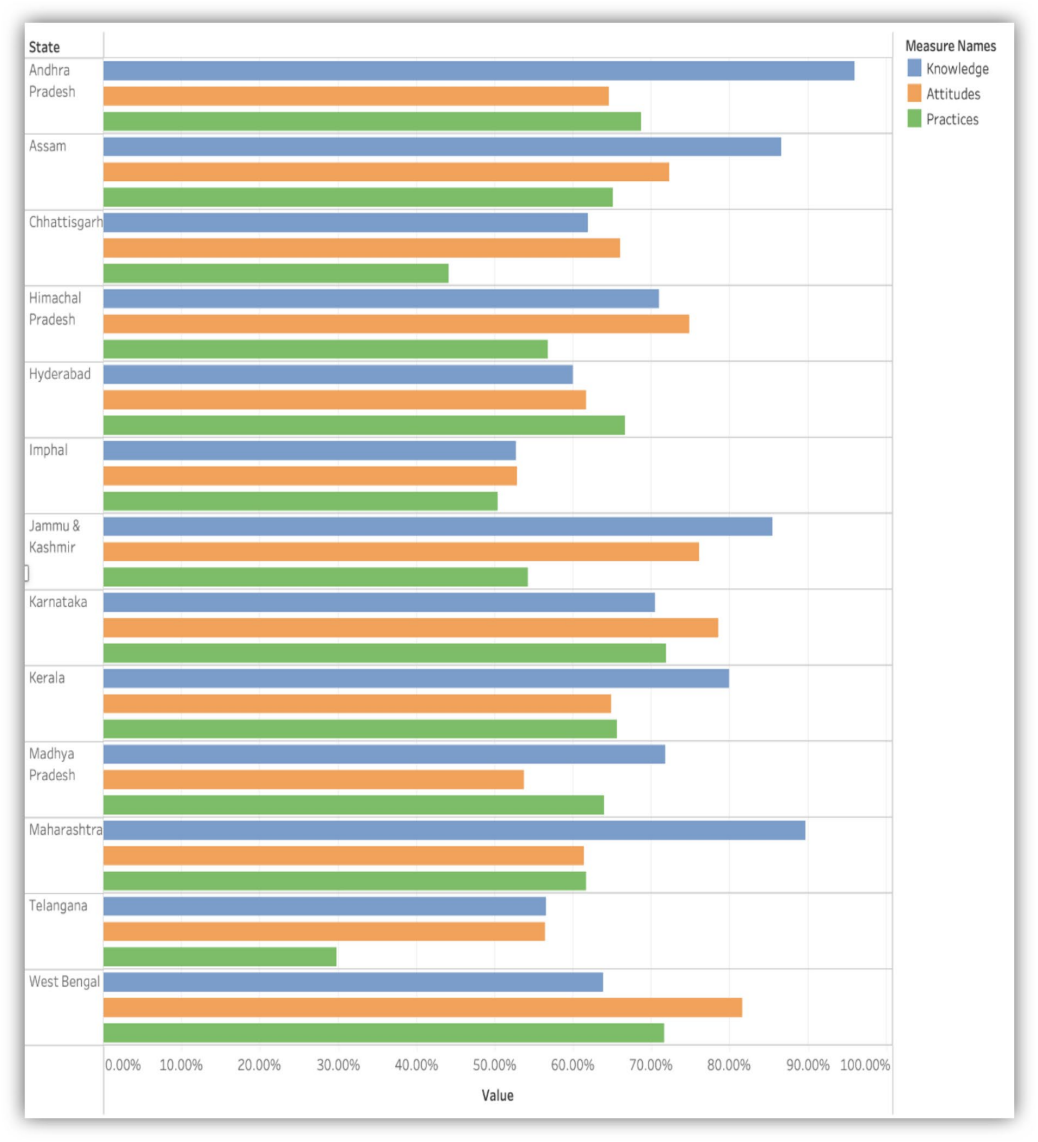
Comparative analysis of knowledge, attitudes, and practices (KAP) regarding antimicrobial resistance among healthcare professionals across 13 Indian states.

Out of the 19 studies initially considered, 17 were included based on their provision of KAP percentages regarding AMR (21–29, 31–36, 38, 39). For the Karnataka and Kerala studies, graphical representation was derived from the average results of three studies each, resulting in a final inclusion of 13 studies for comparative analysis. Findings revealed that Andhra Pradesh has the highest knowledge, followed by Assam, Jammu Kashmir and Maharashtra. West Bengal showed the most positive attitudes at 80%, followed by Karnataka at 78%. For the practices, Karnataka revealed 72% of positive practices, followed by West Bengal at 70% (Figure 7).

## Discussion

The review highlighted the important challenges in combating AMR, which include unrestricted access to antibiotics, inadequate infection prevention control (IPC) measures, and the absence of stringent guidelines to mitigate the emergence of AMR. These findings underscore the urgent need for targeted interventions tailored to address the specific healthcare challenges across different regions of India. Additionally, our analysis revealed the geographic disparities in AMR awareness and practices, as evidenced by studies conducted in varied regions such as West Bengal, Karnataka, and Kerala (21, 23–26, 29, 30, 34, 37). These regional differences underscore the necessity for customized interventions that can effectively address the unique healthcare landscape and challenges in each area.

The study also observed a gap between theoretical knowledge and practical application of AMR principles among healthcare providers in India. Despite a high level of awareness, instances of inappropriate antibiotic prescribing practices, such as incomplete courses and unnecessary usage, were consistently reported. Furthermore, variations in knowledge levels across different healthcare providers were observed, with medical students exhibiting greater awareness likely due to their recent training. It highlights the importance of ongoing training programs for senior faculty members to ensure consistent adherence to best practices.

The study further highlighted the gap between knowledge and attitudes toward AMR, particularly among medical students, who exhibited higher knowledge scores but lower attitudes. Addressing this necessitates the development of methodologies aimed at modifying entrenched individual behaviors and fostering a culture that promotes rational antibiotic use among young medical professionals. The findings revealed a disparity between theoretical knowledge and practical application of AMR principles among healthcare providers in India. Despite a high level of awareness, instances of inappropriate antibiotic prescribing practices, such as incomplete courses and unnecessary usage, were consistently reported. Furthermore, there were variations in knowledge levels across different healthcare providers, with medical students showing greater awareness, likely due to recent training. It underscores the importance of ongoing training programs for senior faculty members to ensure consistent adherence to best practices.

Moreover, the study highlighted a gap between knowledge and attitudes toward AMR, particularly among medical students, who demonstrated higher knowledge scores but lower attitudes. This means that while these students possess the necessary theoretical understanding of AMR, there needs to be more in their willingness to apply this knowledge in practices. To address this issue, it is imperative to develop methodologies aimed at modifying individual behaviors and fostering a culture that promotes rational antibiotic use among young medical professionals. This could involve implementing targeted educational programs, integrating practical training on antibiotic stewardship into medical curricula, and creating supportive environments that encourage adherence to guidelines for antimicrobial prescribing.

Our review’s findings demonstrates that substantial knowledge of AMR does not automatically translate into practical antimicrobial usage, highlighting a gap between knowledge and practices among healthcare professionals (40, 41). Similarly, studies conducted in other countries like Egypt, Jordan, and China have also shown the widespread dissemination of AMR-related misconceptions among medical students and the public (42–44). Therefore, integrating comprehensive AMR curricula into healthcare education is essential to bridge this knowledge gap and instil the necessary attitudes and practices for AMR control.

In our review, Krishna et al. (22) study reported the highest knowledge score of 96.00% among medical students in Andhra Pradesh. In contrast, the study by Sangma et al. (28) reported the lowest knowledge score of 52.69%, highlighting significant regional disparities in AMR awareness. Another study by Thakolkaran et al. (24) showed the highest attitude score of 89.00% among doctors in Karnataka, suggesting a very positive outlook toward AMR management in this region. This contrasts with the findings of Sangma et al. (28), which had the lowest attitudes score of 52.78%. In terms of practices, the study by Asharani et al. (34) reported the highest score of 83.45% among medical students in Karnataka, indicating effective application of AMR knowledge in practice.

Conversely, the study by Tanveer et al. (38) in Telangana reported the lowest practices score of 29.84%, underscoring the variability in practical implementation across regions. The study by Yashin et al. (27) in Assam presented balanced high scores across all KAP domains, reflecting a well-rounded understanding and application of AMR principles among participants. These comparisons with other Indian studies underscore the need for tailored regional strategies to address specific gaps and leverage existing strengths in AMR awareness and practices across India.

In conclusion, our systematic review aligns with global trends and underscores the need for multifaceted approaches to AMR education. By using insights derived from the review, India can lead sustainable initiatives to mitigate the public health threat posed by AMR. It’s crucial to recognize regional differences and implement tailored interventions accordingly. Through collaboration between policymakers, healthcare institutions, and educational bodies, we can effectively promote rational antimicrobial use and protect public health.

## Conclusion

This systematic review highlights the need for a coordinated, multifaceted strategy to combat AMR in India. Given the unique challenges and complexities faced by healthcare systems in developing countries like India, it is essential to integrate educational initiatives and enhanced diagnostic capabilities. Addressing the significant gap between KAP among healthcare providers necessitates a concerted effort from policymakers, educators, and the community. The findings of this review serve as a crucial framework for crafting evidence-based strategies and interventions aimed at enhancing antibiotic prescription practices and curbing the escalating challenge of AMR within the Indian context.

## Recommendation

This systematic review highlights the gaps and regional disparities in the knowledge, attitudes, and practices (KAP) regarding antimicrobial resistance (AMR) among healthcare workers in India. Based on these findings, we recommend the following targeted strategies to address these gaps and improve AMR management across the country.

For government and policymakers, we recommend developing and enforcing comprehensive national guidelines on antibiotic use and AMR management, as well as establishing robust AMR surveillance systems to monitor resistance patterns and inform policy decisions. Healthcare institutions and administrators should standardize AMR training programs for all healthcare workers and implement stringent infection control measures with regular audits. Educational institutions should integrate AMR education into medical and nursing curricula and offer continuous professional development opportunities to keep healthcare professionals updated on AMR issues. Pharmaceutical companies are encouraged to promote the rational use of antibiotics, discourage over-the-counter sales without prescriptions, and invest in the research and development of new antibiotics and alternative treatments. Healthcare workers should adopt best practices based on the latest AMR guidelines and actively educate patients about the importance of adhering to prescribed antibiotic regimens. By fostering a culture of adherence and informed use among patients, healthcare workers can play a crucial role in mitigating the spread of AMR.

By addressing the specific needs and responsibilities of these stakeholder groups, we can create a coordinated and effective response to combat AMR in India. This comprehensive approach will help bridge the gaps identified in our review and enhance the overall quality of AMR management across the country, ultimately leading to better health outcomes and more sustainable use of antibiotics.

### Limitations

There are several limitations in this study. Firstly, our focus on healthcare workers in India may limit the generalizability of our findings to diverse global contexts of AMR. Secondly, our inclusion criteria include only English language publications, which introduce a potential language bias. Additionally, by limiting our analysis to original research articles, we may have missed critical insights provided by reviews and commentaries that could offer a more comprehensive context. Furthermore, the variability in study designs and methodologies across the included articles may have influenced the overall review outcomes. The absence of longitudinal studies impedes our ability to assess long-term trends and the effectiveness of interventions over time. A comprehensive understanding of AMR awareness trajectories and policy effectiveness is crucial for guiding future interventions. Lastly, the possibility of the regional biases in our review as it includes studies across various states across India, with significant representation from regions such as Kerala, Karnataka, West Bengal, and Assam. While this broad geographic distribution allows for a comprehensive understanding of AMR awareness across different parts of the country, it also introduces the possibility of regional biases that could influence the findings. Potential regional biases arise from the varying healthcare infrastructures, educational standards, and socioeconomic conditions across different states. Moreover, cultural differences and local health policies could impact the attitudes and practices of healthcare workers toward AMR. Regions with stronger regulatory frameworks for antibiotic use and better infection control practices may report more favorable attitudes and practices.

Conversely, regions lacking stringent regulations and resources may struggle with higher rates of inappropriate antibiotic use, reflecting negatively on their AMR-related KAP scores. These regional biases have significant implications for the generalizability of the findings. While the high KAP scores from well-represented states can provide valuable insights, they may not fully capture the challenges faced by healthcare workers in less represented or underdeveloped regions. Therefore, it is crucial to interpret the findings with an understanding of these regional disparities.

To mitigate the impact of regional biases, it is imperative that future research aims for a more balanced representation of states, particularly those with varying levels of healthcare development. Additionally, future studies should broaden their inclusion scope to encompass a more diverse range of study types. Efforts should also be made to translate knowledge into practical, actionable strategies within healthcare settings. By addressing these limitations, we can advance our understanding of AMR and develop more effective strategies to combat this pressing global health threat.

## Author contributions

SR: Conceptualization, Data curation, Formal analysis, Methodology, Writing – original draft. KK: Data curation, Writing – review & editing. PN: Writing – review & editing. KW: Writing – review & editing. SS: Writing – review & editing. AC: Writing – review & editing. MS: Supervision, Writing – review & editing. HS: Methodology, Project administration, Supervision, Validation, Writing – review & editing.
